# Inertial Sensor-Based Recognition of Field Hockey Activities Using a Hybrid Feature Selection Framework

**DOI:** 10.3390/s25247615

**Published:** 2025-12-16

**Authors:** Norazman Shahar, Muhammad Amir As’ari, Mohamad Hazwan Mohd Ghazali, Nasharuddin Zainal, Mohd Asyraf Zulkifley, Ahmad Asrul Ibrahim, Zaid Omar, Mohd Sabirin Rahmat, Kok Beng Gan, Asraf Mohamed Moubark

**Affiliations:** 1 Department of Electrical, Electronic and Systems Engineering, Faculty of Engineering and Built Environment, Universiti Kebangsaan Malaysia (UKM), Bangi 43600, Selangor, Malaysia; nasharuddin.zainal@ukm.edu.my (N.Z.); ahmadasrul@ukm.edu.my (A.A.I.); kbgan@ukm.edu.my (K.B.G.);; 2 Department of Biomedical Engineering & Health Sciences, Faculty of Electrical Engineering, Universiti Teknologi Malaysia (UTM), Skudai, Johor Bahru 81310, Johor, Malaysia; 3 Sport Innovation & Technology Centre, Institute of Human Centered Engineering, Universiti Teknologi Malaysia (UTM), Skudai, Johor Bahru 81310, Johor, Malaysia; 4 Department of Mechanical and Manufacturing Engineering, Faculty of Engineering and Built Environment, Universiti Kebangsaan Malaysia (UKM), Bangi 43600, Selangor, Malaysia; hazwanghazali@ukm.edu.my (M.H.M.G.);; 5 Department of Electronic & Computer Engineering, Faculty of Electrical Engineering, Universiti Teknologi Malaysia (UTM), Skudai, Johor Bahru 81310, Johor, Malaysia

**Keywords:** activity recognition, classification, feature selection, human activity recognition, wearable sensor

## Abstract

Accurate recognition of complex human activities from wearable sensors plays a critical role in sports analytics and human performance monitoring. However, the high dimensionality and redundancy of raw inertial data can hinder model performance and interpretability. This study proposes a hybrid feature selection framework that combines Minimum Redundancy Maximum Relevance (MRMR) and Regularized Neighborhood Component Analysis (RNCA) to improve classification accuracy while reducing computational complexity. Multi-sensor inertial data were collected from field hockey players performing six activity types. Time- and frequency-domain features were extracted from four body-mounted inertial measurement units (IMUs), resulting in 432 initial features. MRMR, combined with Pearson correlation filtering (|*ρ*| > 0.7), eliminated redundant features, and RNCA further refined the subset by learning supervised feature weights. The final model achieved a test accuracy of 92.82% and *F*1-score of 86.91% using only 83 features, surpassing the MRMR-only configuration and slightly outperforming the full feature set. This performance was supported by reduced training time, improved confusion matrix profiles, and enhanced class separability in PCA and t-SNE visualizations. These results demonstrate the effectiveness of the proposed two-stage feature selection method in optimizing classification performance while enhancing model efficiency and interpretability for real-time human activity recognition systems.

## 1. Introduction

Human Activity Recognition (HAR) is increasing area within machine learning that presents a wide array of applications across sectors such as healthcare, sports, security, and human–computer interaction. This field leverages sensor data and advanced algorithms to accurately detect and classify various physical activities, from straightforward movements like walking to intricate actions such as dancing or engaging in sports. HAR methodologies are generally classified into two primary categories: vision-based and sensor-based approaches. Vision-based systems employ cameras to monitor movement and assess the surrounding environment; however, they often face challenges including privacy concerns, limitations posed by inadequate lighting, and potential visual obstructions. Moreover, these systems typically necessitate sophisticated processing techniques and costly camera setups to ensure optimal performance [1].

Conversely, sensor-based approaches employ various wearable and environmental sensors, facilitating continuous monitoring of activities and surrounding contexts, which enhances user engagement. A comprehensive survey [2] detailed the evolution of HAR, illustrating a transition from simple task recognition to the ability to identify complex and concurrent activities using sensor-based methodologies. This survey reviewed 190 scholarly articles, examining various machine learning (ML) and deep learning (DL) techniques while identifying significant challenges, including the necessity for personalized models and efficient data collection methods [2]. The crucial role of HAR in healthcare and security is emphasized, highlighting the need for advancements in interpretability through explainable AI (XAI) [2]. Additionally, a study [3] provided valuable guidance for researchers regarding the selection of suitable datasets and methodologies aimed at enhancing HAR accuracy, addressing the challenges posed by the multitude of available datasets and their diverse characteristics. The findings underscore the importance of intentional dataset selection and systematic evaluation processes in advancing HAR research [3].

Nonetheless, wearable systems may face limitations, including user discomfort during wear, restricted sensor coverage, and challenges associated with device positioning and battery life [2]. Furthermore, the necessity for individuals to wear these devices can sometimes feel intrusive, potentially affecting their natural behaviour [2]. Therefore, precise sensor placement and calibration are vital for ensuring accurate recognition [2,4]. The vast array of available datasets complicates the identification of those most appropriate for specific evaluative needs, as datasets often vary regarding the types of activities recorded, the devices used for data collection, annotation quality, and the context of data acquisition. This variation necessitates careful consideration of preprocessing techniques, feature extraction, and selection aligned with the chosen dataset [3].

The potential implications of HAR technology are profound, offering the promise of personalized health monitoring systems capable of continuously tracking and enhancing individual well-being. Furthermore, this technology has the potential to transform sports performance analysis, providing invaluable insights into athlete movements and refining training methodologies as discussed in studies [5,6,7,8]. Advanced surveillance systems could similarly leverage these capabilities to recognize and respond to a broad spectrum of activities in real-time, thereby enhancing public safety [8,9]. These essential devices continuously monitor and record a user’s motion and orientation, generating extensive datasets that capture the nuances of their movements [10]. Following data collection, the information undergoes a critical preprocessing phase, during which it is refined and transformed into meaningful features that are necessary for machine learning models, thus facilitating precise activity classification [10].

Despite significant advancements in HAR, a prevailing challenge endures the management of high-dimensional feature spaces. This complexity arises from the use of multiple sensors that produce diverse and abundant data. While an extensive array of features can improve recognition accuracy, it simultaneously introduces challenges such as increased computational costs, the risk of overfitting, and diminished model efficiency a phenomenon often referred to as the “curse of dimensionality.” Additionally, the inclusion of redundant or irrelevant features can compromise the performance and reliability of the model.

To address this, the present study emphasizes the importance of feature selection and dimensionality reduction to enhance the accuracy, interpretability, and computational efficiency of HAR systems. By reducing the feature set to only the most informative variables, the model can better capture meaningful patterns in human motion. The key contributions of this study are as follows, (1) improved model performance through a hybrid feature selection framework combining MRMR with correlation filtering and RNCA, the feature set was reduced from 432 to 83, leading to higher accuracy and *F*1-score while lowering computational complexity, (2) enhanced model interpretability through proposed method allows identification of the most relevant features, offering insights into which sensor signals are most discriminative for classifying different human activities and (3) reduced redundancy and improved robustness by removing unnecessary features, the model becomes more resilient to noise and performs more consistently across different users and activity conditions.

## 2. Related Works

### 2.1. High-Dimensional Feature Representation

The term “high feature dimension” refers to datasets characterized by a significant number of features or variables, which often results in computational and theoretical complexities [11]. The importance of high-dimensional data [11] has notably increased with the advent of large-scale applications in areas such as bioinformatics, image processing, natural language processing, and sensor-based activity recognition. However, the intricacies associated with high-dimensional data present substantial challenges in the training of machine learning models, the optimization of performance, and the achievement of robust generalization capabilities [12].

A well-documented challenge in high-dimensional spaces is known as the curse of dimensionality [12]. As the number of features escalate, the volume of the feature space expands exponentially, leading to issues of sparsity where data points become increasingly dispersed [11]. This sparsity complicates the process for machine learning models to identify meaningful patterns, as most points in high-dimensional space are often equidistant from one another [12]. Consequently, distance-based learning algorithms, such as k-nearest neighbors (k-NN) and various clustering methods that struggle to differentiate between relevant and irrelevant features, which can adversely affect classification performance and introduce model bias.

Overfitting constitutes another critical concern when working with high-dimensional data [11]. Overfitting occurs when a model becomes excessively complex, capturing noise rather than the underlying patterns present in the dataset. When the number of features is significantly larger than the number of observations, models may demonstrate excellent performance on training data yet fail to generalize effectively to new, unseen instances [13]. Beyond the issue of overfitting, high-dimensional feature spaces also introduce substantial computational challenges. The processing of large datasets comprising numerous features demands considerable computational resources, including elevated memory usage and prolonged processing times.

To mitigate these challenges, researchers commonly utilize feature selection and dimensionality reduction techniques [14]. Feature selection methods aim to identify and retain only the most informative features while discarding those that are redundant or irrelevant. These methods can be generally classified into three categories: filter methods, wrapper methods, and embedded methods [11,15]. Among the various techniques employed for feature selection, the neighbor-based approach is recognized as a particularly effective method for supervised feature learning and dimensionality reduction.

Neighborhood-based methods aim to improve classification performance by adjusting feature representations so that data points from the same class are closer together, while those from different classes are farther apart. One widely used technique in this category is Neighborhood Component Analysis (NCA), which learns a distance metric to enhance class separability. NCA has been applied in various fields, including image recognition and biometric identification, where selecting the right features is essential. However, NCA can be computationally demanding, especially when applied to large datasets due to the need for pairwise distance calculations. Despite this, ongoing research in metric learning continues to improve its efficiency and practical use in machine learning for classification.

### 2.2. Overview of Current RNCA-Based Studies

RNCA is an advancement of NCA that significantly enhances the objective generalization capabilities of the NCA framework. Study by Yang et al. introduced regularization terms into the RNCA methodology, which effectively controls the complexity of the learned projection matrix [16]. This approach is instrumental in mitigating the risk of overfitting by discouraging the adoption of excessively large weight values [16]. Notably, the proposed methods demonstrate insensitivity to the choice of a specific regularization parameter, as evidenced in the formulation presented in (1)

(1) $$\xi (w)=\frac{1}{N}\sum_{i}\sum_{j}^{n}y_{ij}p_{ij}-\lambda \sum_{l=1}^{d}w_{l}^{2}$$

A study in [17] indicated that RNCA serves as an effective regularization technique that strikes a balance between enhancing class separability and mitigating the risk of overemphasis on specific features. This technique is particularly advantageous in high-dimensional datasets, where the propensity for overfitting is markedly heightened. By providing a more controlled approach to dimensionality reduction in conjunction with NCA, RNCA enhances generalization performance across diverse classification tasks. For instance, a study by Kumar et al. [18] implemented regularized feature selection regression based on Neighborhood Component Analysis (FSRNCA) to identify significant predictors of mortality rates. This methodology successfully highlighted essential attributes from the Big Cities Health Inventory dataset, encompassing demographic, socio-economic, and health-related metrics [18].

Despite the advantages of the RNCA approach, the methodology may not sufficiently capture interaction effects among features, thereby neglecting complex interrelationships that could enhance predictive accuracy [18]. Moreover, the characteristics of the dataset may dictate which features are deemed significant which potentially resulting in the exclusion of critical predictors. While dimensionality reduction can facilitate model efficiency, it also poses a risk of discarding valuable information, particularly when features are evaluated in isolation. The effectiveness of the RNCA technique is also contingent upon the quality of the input data deficiencies such as missing or inaccurate values can lead to skewed feature selection outcomes [19]. Lastly, although RNCA is designed to be computationally efficient. The processes involved in identifying optimal parameters and assessing feature importance can still require considerable computational resources, which may not be feasible for extensive datasets or real-time applications [18]. Collectively, these limitations underscore the necessity for meticulous consideration and validation in the application of RNCA to ensure that selected features contribute to constructing robust and precise predictive models.

Recent studies have made notable contributions to the advancement of hybrid feature selection and dimensionality reduction methodologies tailored for classification tasks. One such method, referred to as BAROQUE, integrates bee swarm optimization (BSO) with a multi-agent deep Q-network (DQN) to enhance human activity recognition utilizing wearable sensors [20]. This hybrid approach effectively balances exploration and exploitation within the feature space, facilitating an adaptive and efficient local search process [20]. The advancements significantly enhance the performance and efficiency of activity recognition derived from sensor data [20]. Besides that, Shahar. N et al. [21] introduced the RNCA approach based on (2)

(2) $$I(X,Z)=\sum_{i,j}P(X=x_{i},\;\;Z=z_{j})\;log\frac{P(X=x_{i},\;\;\;Z=z_{j})}{P(X=x_{i},\;\;\;Z=z_{j})}$$

where *I* is a mutual information of the discrete random variables *X* and *Z*. The goal of the MRMR algorithm is to find an optimal set *S* of features that maximizes *V_S_*, the relevance of *S* with respect to a response variable *y*, and minimizes *W_S_*, the redundancy of *S*, where vs. and *W_S_* are defined with mutual information *I*

(3) $$V_{S}=\frac{1}{\left[S\right]}\sum_{x\in S}I(x,y)$$

(4) $$W_{S}=\frac{1}{\left[S\right]2}\sum_{x\in S}I(x,z)$$

|*S*| is the number of features in *S*. Finding an optimal set *S* requires considering all 2^|Ω|^ combinations, where Ω is the entire feature set. Instead, the MRMR algorithm ranks features through the forward addition scheme. This enhances model performance compared to traditional methods through efficient feature selection which transforms the feature space to better separate classes, improving classification in high-dimensional data and addressing overfitting and the result showed the outperforming models based on Original Feature alone [21]. Also study by Chen et al. focused on a feature selection method utilizing Neighborhood Component Analysis (NCA) to identify the most discriminative features from extracted feature vectors [22]. This approach effectively reduced dimensionality while maintaining essential information, ensuring that only the most relevant features were utilized in the k-nearest neighbors (kNN) classifier, thereby enhancing classification performance [22].

RNCA technique was implemented after the attention layer of a deep convolutional neural network, enhancing model performance by emphasizing the most relevant features derived from the mel-spectrogram [23]. The incorporation of RNCA resulted in a 3.7% increase in accuracy, culminating in an overall classification accuracy of 97.8% [23]. This finding underscores the critical role of feature selection in enhancing the discriminative power of models within emotion recognition tasks [23]. Moreover, Sahu and Ray introduced an innovative approach for the detection and localization of myocardial infarction (MI) by integrating Variational Mode Decomposition (VMD) with RNCA to analyze ECG signals [24]. In [24], KNN and AdaBoost classifiers achieved an impressive accuracy of 99.82% in MI detection using just 33 features from specific lead data and successfully classified with an accuracy of 99.75% using 22 features from another lead [24]. This illustrates the potential for real-time applications in portable health monitoring [24].

Identifying the optimal feature set for HAR poses a considerable challenge. The advantages of feature selection in machine learning are extensive, including enhanced learning performance, characterized by higher accuracy and lower computational costs as well as improved model interpretability [25,26,27]. RNCA addresses several issues related to feature selection but has limitations, particularly in modeling non-linear relationships and managing redundant features in high-dimensional spaces. Shahar et al. [21] proposed an optimal activity recognition Framework integrating MRMR with RNCA, which improved classification accuracy and dimensionality reduction. However, this framework did not account for residual multicollinearity among highly ranked MRMR features, complicating models and reducing interpretability.

To address this gap, the current study introduces a Pearson correlation-based filtering step post-MRMR ranking, removing highly correlated features with a threshold of |*p*| > 0.7. This refinement results in a more independent feature subset, enhancing interpretability and computational efficiency. Furthermore, the study employs comprehensive statistical evaluations, including paired two-tailed *t*-tests, 95% confidence intervals, and confusion matrix analyses. Dimensionality reduction techniques, such as PCA and t-Distributed Stochastic Neighbor Embedding (t-SNE), are utilized to visualize class separability.

In summary, incorporating Pearson correlation filtering into the MRMR-RNCA pipeline significantly enhances model compactness, generalizability, and computational efficiency, rendering it suitable for real-time, sport-specific HAR classification critical for performance monitoring and athlete safety.

## 3. Methods

### 3.1. Field Hockey Dataset Acquisition

The dataset utilized in this study was originally acquired and pre-processed as part of the authors’ prior study [21]. It comprises inertial sensor-based human activity data collected from 43 healthy participants (aged 13–20 years), including both male and female individuals. All participants were amateur or semi-professional field hockey players and reported no musculoskeletal injuries during the time of data acquisition. The experimental protocol was designed to capture realistic, sport-specific movement patterns across a predefined set of six field hockey actions: passing, driving, drag flicking, dribbling, receiving, and hitting. Each participant was instrumented with four wearable inertial measurement units (IMUs) (Physilog^®^ 5—Motion Sensor, Physilog, Lausanne, Switzerland) securely mounted using elastic Velcro straps at the chest, waist, left wrist, and right wrist. Each IMU recorded signals from a triaxial accelerometer and a triaxial gyroscope, resulting in six channels per sensor: acceleration along the *X*, *Y*, and *Z* axes (*ACCX*, *ACCY*, *ACCZ*), and angular velocity along the same axes (*GYROX*, *GYROY*, *GYROZ*). All signals were sampled at a frequency of 128 Hz. At any given time step t the raw sensor readings from sensor i∈{1, 2, 3, 4} were defined as:

(5) $${X\left(t\right)}_{i}\;=\left\{ACCXi\left(t\right),\;ACCYi\left(t\right),\;ACCZi\left(t\right),\;GYROXi\left(t\right),\;GROYi\left(t\right),\;GYROZi\left(t\right)\right\}$$

where i = 1 to 4 ccorresponds to the sensor locations (chest, right wrist, waist, and left wrist, respectively). Signals from all four sensors were concatenated to form a 25-dimensional feature vector at each time step, with 24 sensor-derived features and a 25th element indicating the class label Y∈{1, 2, 3, 4, 5, 6}. Each class label corresponded to one of the six field hockey activity categories. To ensure precise and reliable activity annotation, all data collection sessions were video-recorded, and labels were manually assigned using ELAN 5.7 software, with timestamps synchronized to the IMU recordings. This labeling approach ensured high-quality annotations and facilitated subject-specific validation in a controlled, outdoor sports environment. In this study, the dataset was used to evaluate the performance of different feature configurations, each representing a distinct stage in the feature selection pipeline, (1) original Feature Configuration: Consisted of 432 raw extracted features without any reduction, (2) MRMR Configuration: A reduced feature set obtained using MRMR followed by correlation-based filtering and (3) RNCA Configuration: A compact feature set refined from the MRMR output using RNCA.

### 3.2. Window Segmentation

For feature extraction and classification, a fixed-length sliding window segmentation approach was applied to each subject’s continuous signal recordings. This method preserves local temporal dependencies while structuring the data into manageable units for downstream analysis. For each subject, the raw dataset $X\left(t\right)\in R^{n\;X\;25}$, where n denotes the number of time steps and 25 represents the 24 sensor features plus one class label, was segmented into windows of fixed length *W* = 141-time steps. This window size, equivalent to approximately 1.1 s of activity at a sampling rate of 128 Hz, was adopted based on prior work [21] to ensure sufficient temporal resolution for activity recognition. To maintain continuity and capture transitional motion patterns, 50% overlap was applied between successive windows, corresponding to a stride of 70-time steps and an overlap size of O=71. The total number of segments *k* generated from each subject’s time series was calculated as:

(6) $$k=\left\lfloor \frac{n-W}{W-O}\right\rfloor +1$$

Each window $W_{j}$, j=1, 2, …,k was defined as a continuous sequence of samples:

(7) $$W_{j}\;=\{X(t_{j}),\;X(t_{j}+1),\ldots X(t_{j}+W-1)\}$$

where the starting time index for each, j-th window given by:

(8) $$t_{j}=j.(W-O)$$

Each resulting segment is a matrix of size W × 25, containing 24 sensor features across all four IMUs and one class label. The class label $Y_{j}$ for window $W_{j}$ was assigned using majority voting over the class labels within the windowed interval. In instances where class transitions occurred within a window, the most frequent (dominant) class label was selected to ensure consistency in labeling. This overlapping segmentation scheme improves model robustness by reducing sensitivity to window boundary artifacts and by capturing finer-grained variations in movement patterns. Moreover, it allows the classifier to learn localized temporal dynamics critically for distinguishing between complex, dynamic human activities.

### 3.3. Time and Frequency Feature Extraction

A comprehensive set of statistical and frequency-domain features was extracted from each segmented window to capture the underlying characteristics of human motion. Each window $W_{j}\in R^{141\;X\;25}$, corresponding to approximately 1.1 s of activity, comprised 24 sensor signal channels and one class label. For each of the four wearable sensors, six channels were recorded: three-axis acceleration (*ACCX*, *ACCY*, *ACCZ*) and three-axis angular velocity (*GYROX*, *GYROY*, *GYROZ*). Feature extraction was conducted independently for each channel and each sensor. In total, 18 features were computed per signal channel, comprising 14 time-domain and 4 frequency-domain features. This yielded a total of 4 sensors × 6 channels × 18 features = 432 features per window, referred to as the Original Feature Configuration.

The time-domain features were selected to describe the distributional, statistical, and dynamic properties of each signal. Let $x_{j}(i)$ represent the value of signal dimension j at time step *i* within a window of length N=141. The following 14 features were computed:

(9) $$f_{\mu }^{\left[j\right]}=\frac{1}{N}\sum_{i=1}^{N}x_{j}(i)$$

(10) $$f_{\sigma }^{\left[j\right]}=\sqrt{\frac{1}{N-1}\sum_{i=1}^{N}{|x_{j}(i)-\mu |}^{2}}$$

(11) $$f_{\max}^{\left[j\right]}=\max x_{j}(i)$$

(12) $$f_{\min}^{\left[j\right]}=\min x_{j}(i)$$

(13) $$f_{v}^{\left[j\right]}=\frac{1}{N-1}\sum_{i=1}^{N}{|x_{j}(i)-\mu |}^{2}$$

(14) $$f_{\mathrm{median}}^{\left[j\right]}=x_{j}\left(i\right)[\left(\frac{N+1}{2}\right)]$$

(15) $$f_{MDF}^{\left[j\right]}\;\mathrm{represented}\;\mathrm{by}\;\mathrm{the}\;\mathrm{most}\;\mathrm{frequent}\;\mathrm{value}$$

(16) $$f_{\mathrm{range}}^{\left[j\right]}=f_{\max}^{\left[j\right]}-f_{\min}^{\left[j\right]}$$

(17) $$f_{\mathrm{kurtosis}}^{\left[j\right]}=\frac{1}{N}\sum_{i=1}^{N}\frac{{\left(x_{j}(i)-µ\right)}^{4}}{\sigma^{4}}$$

(18) $$f_{skw}^{\left[j\right]}=\frac{1}{N}\sum_{i=1}^{N}\frac{{\left(x_{j}(i)-µ\right)}^{3}}{\sigma^{3}}$$

(19) $$f_{m}^{\left[j\right]}=\frac{N}{\sum_{i=1}^{N}\frac{1}{x_{j}(i)}}$$

(20) $$f_{rms}^{\left[j\right]}=\sqrt{\frac{1}{N}\sum_{i=1}^{N}{{[x}_{j}(i)]}^{2}}$$

(21) $$f_{IQR}^{\left[j\right]}=Q_{3}-Q_{1}$$

(22) $$f_{MAD}^{\left[j\right]}=\frac{\sum_{i=1}^{N}\left|x_{j}(i)-µ\right|}{N}$$

These features capture various properties such as central tendency, spread, shape, symmetry, and energy of the signal within each window, offering valuable insights into motion patterns. To complement the time-domain descriptors, each signal was also transformed into the frequency domain using the Fast Fourier Transform (FFT). From the resulting power spectral density, the following four features were extracted:

(23) $$f_{MNP}^{\left[j\right]}=\sum_{i=1}^{N}\frac{x_{j}(i)}{N}$$

(24) $$f_{MNF}^{\left[j\right]}=\frac{\sum_{i=1}^{N}fx}{\sum_{i=1}^{N}f}$$

(25) $$f_{MDF}^{\left[j\right]}=\frac{1}{2}\sum_{i=1}^{N}x_{j}(i)$$

(26) $$f_{BW}^{\left[j\right]}\;\mathrm{represented}\;\mathrm{by}\;\mathrm{the}\;\mathrm{frequency}\;\mathrm{range}$$

These frequency-domain features reflect oscillatory behavior, spectral distribution, and rhythmic components of the signal particularly important for recognizing activities involving repetitive motion, for instance, dribbling. In total, the 432-dimensional feature vector formed the Original Feature Configuration, serving as input for downstream feature selection and classification. This high-dimensional representation was designed to capture a broad range of motion characteristics, enabling accurate discrimination between the six predefined field hockey activity classes.

### 3.4. Feature Selection by MRMR

Due to the high dimensionality of the extracted feature space, comprising 432 features per window, feature selection was deemed essential to eliminate redundancy, enhance computational efficiency, and support better generalization of the classification model. To address this, a two-stage feature selection strategy was employed. In the first stage, the MRMR algorithm was applied to rank features based on their mutual relevance to the class labels and their redundancy with respect to other features. MRMR works on the principle of maximizing the information gain associated with a feature while minimizing its overlap with other features, thereby optimizing the balance between informativeness and diversity. Specifically, for a given feature $f_{i}$, its MRMR score is computed as:

(27) $$\mathrm{MRMR}(f_{i})=I(f_{i}:y)\;-\;\frac{1}{S}\sum_{f_{j}\in S}^{t_{j}+W-1}I(f_{i}:f_{j})$$

where $I\left(f_{i}:y\right)\;$ denotes the mutual information between feature $f_{i}$ and the target class y, representing the feature’s relevance to the classification process. Conversely, $I(f_{i}:f_{j})$ measures the redundancy between $f_{i}$ and another feature $f_{j}$ already included in the selected subset S. The MRMR algorithm proceeds in a sequential manner, selecting features iteratively by maximizing the MRMR score at each step. This ensures that each newly selected feature contributes complementary information while maintaining minimal redundancy with the existing feature set. To further enhance the diversity and discriminative quality of the selected features, a Pearson correlation-based filtering step was applied subsequent to the MRMR ranking. This additional filtering stage discards features that exhibit strong linear correlations with others, even if they were highly ranked by MRMR. The rationale is to eliminate redundancy not fully captured by mutual information alone and to promote a more decorrelated and representative subset. The Pearson correlation coefficient between two features $f_{i}$ and $f_{j}$ was computed using the standard formulation:

(28) $$p_{ij}=\;\frac{cov(f_{i},\;f_{j})}{\sigma_{X_{i}}\sigma_{X_{j}}}$$

where $cov(f_{i},\;f_{j})$ denotes covariance between $f_{i}$ and $f_{j}$, while $\sigma_{X_{i}}$ and $\sigma_{X_{j}}$ represent the standard deviations. Following the initial MRMR ranking, a correlation threshold of |*ρ*| = 0.7 was applied to identify and eliminate highly redundant features exhibiting strong linear dependence. To reduce redundancy among the extracted features, a correlation threshold of |*ρ*| = 0.7 was applied following the initial MRMR ranking. This threshold is widely used in literature as a pragmatic compromise between retaining relevant signal variation and minimizing multicollinearity. However, as emphasized by Schober et al. [28], the interpretation of correlation strength is inherently context-dependent, and fixed cut-off values should be viewed as heuristics rather than rigid statistical boundaries. In [28], the discussion on correlation coefficients has provided a commonly cited interpretive scale but caution against over-reliance on arbitrary thresholds without considering the objectives and data characteristics of the study. In this work, the |*ρ*| = 0.7 threshold was adopted not as an absolute cutoff, but as a task-specific heuristic aligned with the goals of improving model stability, interpretability, and computational efficiency in high-dimensional inertial sensor-based classification tasks. In each such condition, the lower-ranked feature (based on MRMR score) was discarded to preserve the more informative counterpart. This correlation filtering step ensured that the resulting subset referred to as the MRMR configuration was not only relevant to the classification task but also minimally redundant and statistically diverse. Such decorrelation enhances the discriminative power of the feature set, reduces the risk of overfitting, and improves model stability particularly in distance-based classifiers such as k-Nearest Neighbors (k-NN), where collinear features can distort distance metrics and degrade performance. Additionally, reducing multicollinearity enhances model interpretability, as each retained feature is more likely to contribute unique and complementary information.

Overall, the application of the |*ρ*| = 0.7 threshold represents a pragmatic and empirically supported strategy for improving model generalization and computational efficiency in high-dimensional, sensor-based HAR. The refined MRMR configuration was subsequently used as input for the second stage of feature refinement via RNCA, which further optimized the subset through a supervised learning criterion.

### 3.5. Feature Selection by RNCA

Following the initial feature dimensionality reduction achieved through MRMR and correlation-based filtering, a second-stage refinement was performed using RNCA. In contrast to MRMR, which evaluates features independently based on mutual information with the target variable, RNCA is a supervised embedded feature selection method that jointly optimizes feature importance weights within the context of a classification task. This approach allows the model to capture feature interactions and to adaptively emphasize features that contribute most effectively to class separation boundaries. Given the MRMR feature matrix ${F\in R}^{nxd}$ where *n* is the number of samples and *d* is the number of selected features and the corresponding label vector ${Y\in N}^{n}$, RNCA seeks to learn a non-negative weight vector ${w\in R}^{d}$ where each component $w_{i}$ quantifies the relative importance of feature the importance of feature i  in minimizing classification loss. The objective of RNCA is to optimize a weighted distance metric such that samples belonging to the same class are pulled closer in the transformed space, while those from different classes are pushed apart. The optimization is formulated to minimize a regularized loss function that balances the classification objective with a penalty term to prevent overfitting:

(29) $$L(w)=\;\sum_{i\;=1}^{n}\sum_{j\neq i\;}p_{ij}.\lambda (f_{i}:f_{j})$$

where $p_{ij}$ denotes the probability of sample i being classified as sample j, based on their distance in the learned, the weighted feature space. The term $\lambda (f_{i},\;f_{j})$ represents a regularization term that penalizes complex or overfit models by discouraging overreliance on a small subset of features. The vector ${w\in R}^{d}$ contains the feature weights to be learned, with each $w_{i}$ reflecting the importance of the corresponding feature $f_{i}$, in minimizing classification loss. To identify an optimal balance between model complexity and generalization performance, cross-validation was employed within the training set to determine the regularization parameter *λ*. This parameter governs the trade-off between fidelity to the training data and resistance to overfitting. A grid search was conducted to select the value of *λ* that minimized validation loss, ensuring that the learned weights generalize well beyond the training data. Once the model was trained, features whose weights exceeded a predefined threshold *τ* were retained for the final model. This thresholding process eliminated features with negligible contributions to class discrimination, resulting in a compact, refined subset of informative features.

A 80/20 train-test split was adopted, whereby the final model was evaluated on unseen data. Importantly, internal cross-validation was restricted to the training set to prevent information leakage and to maintain a valid assessment of model generalization. The output of the RNCA stage is a weighted feature matrix ${F\in R}^{nxd}$, paired with the label vector ${Y\in N}^{n}$, collectively forming the dataset D={F,Y} used for final classification. The strength of RNCA lies in its ability to model joint dependencies among features and to emphasize context-sensitive information capabilities that complement the filter-based MRMR approach used in the first stage. Together, these two stages yield a highly discriminative and compact feature subset, referred to as the RNCA configuration.

### 3.6. Classification and Evaluation

The classification performance of each feature configuration was evaluated using a fine-grained k-Nearest Neighbor (k-NN) classifier with *k* = 1. This non-parametric algorithm was selected for its simplicity, interpretability, and robustness in handling high-dimensional feature spaces properties that are particularly advantageous in wearable sensor-based HAR. To extend the k-NN classifier to multi-class classification, it was embedded within an Error-Correcting Output Codes (ECOC) framework. ECOC decomposes a multi-class task into a set of binary classification problems, each of which is trained independently using the base learner.

In addition, several deep learning classifiers were implemented and trained on the handcrafted feature sets generated from the three configurations using the same feature representation space. This approach ensured that all models were evaluated under consistent input conditions. Thereby, this is purposely to isolate the effect of the proposed hybrid feature-selection pipeline rather than introducing an entirely new representation-learning paradigm. Each feature matrix was normalized using z-score normalization, where the mean and standard deviation were computed from the training partition and subsequently applied to the validation and testing partitions. Three representative deep learning architectures were selected to reflect commonly used paradigms in HAR:

- Multilayer Perceptron (MLP) comprising two fully connected layers (256 and 128 neurons) with ReLU activations and dropout (rate = 0.3) for regularization
- One-dimensional Convolutional Neural Network (1D-CNN) that treated each feature vector as a short temporal sequence, consisting of two convolutional blocks (64 and 128 filters, kernel size = 3) followed by batch normalization, ReLU activation, global average pooling, and a softmax output layer
- Long Short-Term Memory (LSTM) network configured with 64 hidden units and a final dense softmax layer, modelling sequential dependencies across the feature indices.

All networks were trained using the Adam optimizer with a learning rate of 0.001, categorical cross-entropy loss, mini-batch size of 64, and a maximum of 50 epochs. Early stopping was applied based on validation performance to prevent overfitting and L2 regularization was included to stabilize training. The same 80/10/10 split ratio as used in the conventional pipeline was maintained for training, validation, and testing. To mitigate randomness in data partitioning and weight initialization, the entire experimental process was repeated ten independent times, and the results were reported as the mean ± standard deviation across runs. These baselines provided a consistent reference for assessing whether more expressive deep learning classifiers offered measurable advantages within the same handcrafted feature space. It is important to note that end-to-end deep learning directly on raw IMU sequences was not included in this study. As such models address a fundamentally different study problem centered on representation learning rather than feature selection.

The ECOC architecture also provides modularity and scalability, allowing seamless integration with various base classifiers while preserving interpretability and generalization performance. In this study, ECOC was coupled with 1-NN as the base learner. For evaluation, the full dataset was randomly partitioned into 80% training, 10% validation, and 10% testing subsets across ten independent runs, with performance metrics averaged to reduce sampling bias and assess stability. Within each training set, 10-fold cross-validation was performed, and the fold yielding the lowest classification loss was selected. The corresponding trained sub-model was then used to predict both validation and testing subsets, enabling robust model selection and minimizing overfitting. Model performance was primarily evaluated using classification accuracy and the macro-averaged *F*1-score. The *F*1-score was calculated from precision and recall as follows:

(30) $$\mathrm{Accuracy}=\frac{TP+TN}{TP+TN+FP+FN}$$

(31) $$\mathrm{Precision}=\frac{TP}{TP+FP}$$

(32) $$\mathrm{Recall}=\frac{TP}{TP+FN}$$

(33) $$F1\;\mathrm{Score}=\frac{2\;X\;\mathrm{Precision}+\mathrm{Recall}}{\mathrm{Precision}+\mathrm{Recall}}$$

where TP, TN, FP and FN represent the true positives, true negatives, false positives, and false negatives, respectively. *F*1-scores were computed on a per-class basis and then averaged (macro-*F*1) to provide a balanced view of performance across all activity classes, regardless of class frequency. In addition to these metrics, confusion matrices were computed for both training and testing phases to provide class-specific insights into correctly and incorrectly classified instances. This allowed further analysis of class overlaps, model consistency, and potential overfitting behaviors.

### 3.7. Statistical Evaluation and Model Comparison—Paired t-Tests

For each feature configuration, classification experiments were repeated across ten independent runs, with the dataset randomly partitioned into 80% training, 10% validation, and 10% testing in each run. To evaluate the statistical significance of observed differences in classification performance, paired two-tailed *t*-tests were conducted on the test accuracies obtained across runs. The following pairwise comparisons were performed, Original Feature vs. MRMR, Original Feature vs. RNCA, and MRMR vs. RNCA. In each case, the null hypothesis stated that there was no significant difference in the mean test accuracy between the two compared models. A significance level of *α* = 0.05 was adopted.

In addition to hypothesis testing, 95% confidence intervals (CIs) were computed for the mean test accuracy of each configuration to quantify the range within which the true performance is expected to lie. The confidence interval was estimated using the *t*-distribution, according to the following formulation:

(34) $$CI=\;x\;\pm \;t_{\left(1-\frac{\alpha }{2},n-1\right)}.\;\frac{s}{\sqrt{n}}$$

where *x* denotes the mean accuracy, *s* is the standard deviation across the runs, *n* is the number of repetitions and $t_{\left(1-\frac{\alpha }{2},n-1\right)}$ is the critical value from the *t*-distribution with *n* − 1 degrees of freedom. These confidence intervals provide a statistically grounded estimate of the range within which the model’s true test accuracy is likely to fall, accounting for variability due to data partitioning and random sampling effects. Together with the *p*-values from the *t*-tests, CIs offer a robust basis for comparing model generalization performance and stability across different feature selection configurations.

### 3.8. Statistical Evaluation and Model Comparison—Correlation Heat Map

The degree of inter-feature redundancy within each feature configuration was generated using Pearson correlation heatmaps. Pairwise correlation coefficients were computed for all selected features using the Pearson method, resulting in a correlation matrix that was visualized as a heatmap. This analysis provides an interpretable overview of the linear dependencies and potential multicollinearity among features. In the Original Feature configuration, significant clustering of high correlation values was observed, indicating substantial redundancy. In contrast, the MRMR and RNCA configurations exhibited substantially reduced correlation patterns, reflecting the effectiveness of the two-stage feature selection pipeline in eliminating redundant information and retaining statistically diverse, informative features. The correlation heatmap thus served as a qualitative validation tool to assess how well the feature selection methods reduced linear dependence across the retained subset.

Next, the structure of the feature space and the class-wise separability of the data was further investigated using two dimensionality reduction techniques were applied: PCA and t-SNE. PCA projected the high-dimensional features onto a two-dimensional linear subspace by identifying orthogonal axes that captured the maximum variance in the data. This technique facilitated global visualization of class clusters and variance spread. In contrast, t-SNE, a non-linear manifold learning algorithm, was employed to map the high-dimensional feature vectors into a two-dimensional space while preserving local neighborhood structures. This enabled more nuanced visualization of inter-class boundaries and cluster compactness, particularly useful for understanding local class separability.

## 4. Result and Discussion

### 4.1. Feature Selection by MRMR

**Figure 1:**
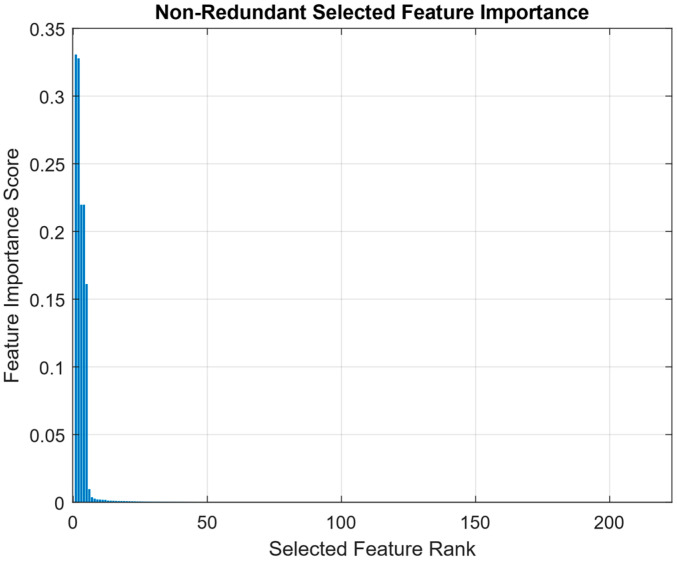
Non-redundant selected feature importance by MRMR.

Figure 1 presents the ranked feature importance scores obtained using the MRMR method following correlation-based filtering. The distribution exhibits a distinctly right-skewed shape, reflecting the discriminative sparsity often found in high-dimensional inertial sensor data for HAR. This skewness indicates that a small subset of features accounts for most of the predictive power, while the majority contribute marginally or redundantly. The curve shows a sharp initial decline in importance, confirming that only a limited number of features are highly relevant to classification. This is followed by a long tail of low-ranked features whose individual importance is minimal. Such a pattern reinforces the necessity of feature selection to mitigate redundancy, reduce overfitting risk, and enhance computational efficiency in HAR classification.

The presence of inflection points and plateaus along the ranking curve suggests a hierarchical structure in feature utility. Top-ranked features likely capture primary biomechanical cues such as dominant limb movements that critically for distinguishing sport-specific actions. In contrast, mid-ranked features may encode subtler patterns or transitional states, while lower-ranked features may reflect noise, irrelevant motions, or correlated signals already represented by higher-ranking ones. Notably, the long-tailed portion of the curve comprises the majority of features, many of which offer limited standalone value. While some may still contribute when considered in combination, their low MRMR scores indicate weak individual discriminative power. This justifies the application of a correlation threshold to filter out redundant or collinear features and retain a more compact, informative subset.

Figure 1 highlights the benefit of a two-stage feature selection strategy. MRMR effectively prioritizes features based on relevance and redundancy, providing an interpretable reduction of the Original Feature space. However, its inability to model joint dependencies or class-conditional relevance motivates the use of a second stage supervised method such as RNCA to further refine the feature set based on predictive performance

Table 1 lists the top 10 features identified through the MRMR algorithm after correlation-based filtering. These features received the highest relevance scores with respect to their ability to discriminate between different field hockey activities. The ranking highlights a strong preference for gyroscope-derived features, especially those capturing rotational dynamics in the upper limbs and torso. This finding aligns well with the biomechanical characteristics of the field hockey activities, where complex upper-body movements such as dribbling, drag flicking, and passing play a dominant role.

The highest-ranked features include statistical descriptors such as range, mean, variance, and median, calculated over specific sensor axes and locations. Several of these top features are derived from the *Z*-axis gyroscope signals of the wrist sensors, indicating that rotational motion particularly around the vertical axis is highly informative for activity differentiation. Features related to vertical acceleration from the arms and torso also appear prominently in the top ranks, suggesting that acceleration patterns in the vertical plane are key discriminators for body posture and movement transitions during gameplay.

A notable observation is that the top five features account for a disproportionate share of the total importance score, consistent with the sharp initial slope observed in Figure 1. This concentration of relevance supports the argument that a small number of highly informative features can effectively drive classification performance, reducing the need to retain all 432 original features. The presence of frequency domain features for instance, power bandwidth and harmonic mean in the later part of the top 10 suggests that spectral content also contributes to activity discrimination albeit to a lesser extent than time-domain statistics. These features may be particularly useful for capturing repetitive motions or intensity variations, especially in activities like dribbling that involve periodic movement cycles.

While MRMR is a univariate selection method that does not consider inter-feature interactions, the resulting top 10 features demonstrate biomechanical plausibility and reflect distinct signal patterns relevant to field hockey. However, the sharp drop in importance after the fifth-ranked feature suggests that subsequent features may provide diminishing returns when used in isolation. Therefore, to further refine the feature set and capture nonlinear or synergistic effects, an embedded supervised method such as RNCA is used in the next stage.

### 4.2. Feature Selection by RNCA

**Figure 2:**
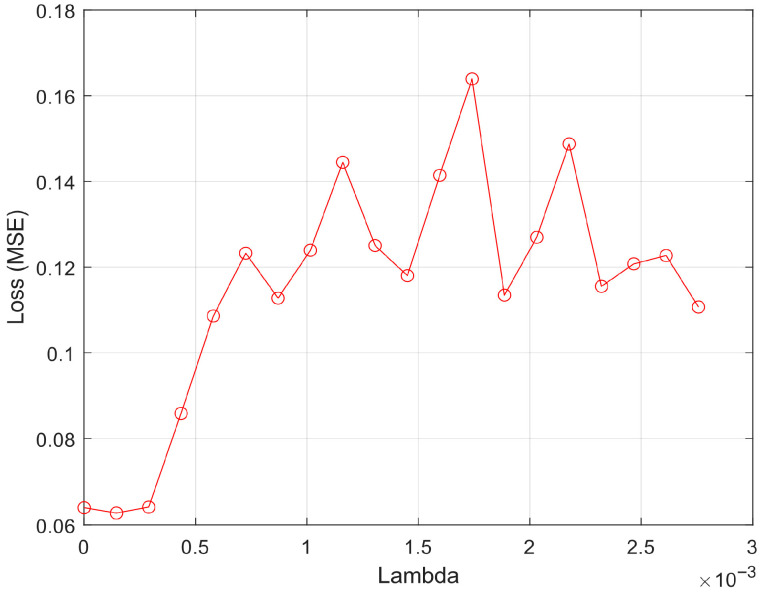
Regularized of λ vs. loss.

Figure 2 illustrates the effect of the regularization parameter (λ) on model performance during the RNCA process. The *x*-axis denotes the range of λ values tested, while the *y*-axis shows the corresponding mean squared error (MSE) on the validation set. Each point represents the loss value obtained from a specific λ, used to assess the model’s generalization capacity. The tuning curve exhibits a clear minimum at λ = 0.000145, where the model achieves its lowest validation loss (MSE = 0.0627). This point represents the optimal trade-off between model complexity and performance, indicating that the selected subset of features at this λ offers the best generalization without overfitting. The gradual rise in MSE on either side of this optimal point suggests that both under-regularization (λ too small) and over-regularization (λ too large) degrade model performance.

Notably, the flat trend near the lower end of λ values (λ < 0.0005) implies that the model is relatively stable and not highly sensitive to small variations in regularization. This stability can be attributed to the effectiveness of the prior MRMR filtering step, which removed most redundant and irrelevant features, thereby reducing the burden on RNCA to compensate for noise or collinearity. The sharp increase in MSE for λ > 0.001 indicates that excessive regularization leads to aggressive penalization of feature weights, effectively shrinking most of them toward zero and suppressing valuable discriminative signals. This behavior underscores the necessity of carefully selecting λ to retain meaningful features while avoiding unnecessary complexity.

Figure 2 supports the validity of the two-stage feature selection pipeline: MRMR ensures feature diversity and relevance, while RNCA fine-tunes the feature weights to minimize error. The low optimal λ value further suggests that the role of RNCA is more about subtle refinement and weighting rather than bulk feature elimination, which is appropriate in high-dimensional, sensor-rich domains such as field hockey activity recognition.

**Figure 3:**
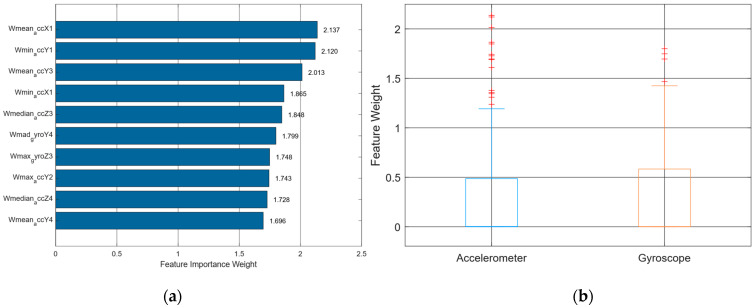
(**a**) Top 10—features vs. weight and (**b**) Box plot of accelerometer and gyroscope.

Meanwhile, Figure 3a presents a horizontal bar chart showing the top 10 features ranked by importance weights as determined by RNCA. Each bar represents a feature’s contribution to the classification model, where a higher weight signifies greater relevance in minimizing classification error. Notably, the top three features, Wmean_accX1, Wmin_accY1, and Wmean_accY3 carry weights exceeding 2.0, clearly dominating the distribution. This indicates a strong preference for acceleration-based features, particularly along the *X* and *Y* axes, which correspond to forward-backward and side-to-side linear movements, respectively.

In contrast to the MRMR selection, which emphasized gyroscopic rotational dynamics, RNCA prioritizes translational movement patterns, suggesting that linear displacement information captured by accelerometers plays a more decisive role in distinguishing activity types during field hockey. This could be due to the positional nature of the target activities, where changes in body location for instance, sprinting during tackling, stopping, passing outweigh rotational motions in classification significance.

Remarkably, only one gyroscope-derived feature, Wmad_gyroY4, with a weight of 1.799 which appears in the top 10. This feature captures median absolute deviation, a robust measure of variability less sensitive to noise, which highlights the preference of RNCA for noise-resistant features from gyroscopes. This aligns with the concept that gyroscopic signals, while useful, are more prone to sensor drift and noise, particularly in dynamic sporting environments. The remaining features in the top 10 include median, minimum, and maximum values, emphasizing that simple statistical descriptors remain highly effective in modeling movement patterns.

Figure 3b complements this analysis by visualizing the distribution of feature weights across the two sensor modalities accelerometers and gyroscopes via a box plot. The distribution shows that accelerometer features exhibit both a higher median and a wider spread of weights, with numerous high-weight outliers. This further underlines the dominance of accelerometer-based features in the final model configuration after RNCA refinement. On the other hand, gyroscope features exhibit a more compact and lower-weight distribution, reinforcing their secondary role in the model. This divergence may reflect both the biomechanical nature of field hockey activities which emphasize locomotion over rotation and sensor-specific properties, such as the relative stability of accelerometers compared to gyroscopes during high-speed movement.

Together, Figure 3a and Figure 3b illustrate that RNCA performs nuanced re-evaluation of feature utility, moving beyond mutual information (as in MRMR) to identify features that directly contribute to reducing classification error. This shift in feature modality from gyroscope to accelerometer, as well as the preference for robust and interpretable features, provides critical insight for future HAR systems in sports contexts. Specifically, these results suggest that emphasizing accelerometer placement and calibration, especially along the *X*-axis, may yield more efficient and interpretable models.

### 4.3. Accuracies Comparison

Table 2 summarizes the classification results across three feature configurations: Original Feature (432), MRMR (222), and RNCA (83). Each configuration was evaluated based on training, validation, and testing accuracies, as well as macro-averaged *F*1 scores, across ten independent experimental runs with random data partitions. The Original Feature configuration, which utilized all 432 extracted features, yielded a training accuracy of 92.32% and a testing accuracy of 92.24%, with an *F*1-score of 85.84%. This performance established a strong baseline, demonstrating the richness and expressiveness of the Original Feature set. However, it came at the cost of increased computational load and risk of overfitting. This is reflected in the broader 95% confidence interval for test accuracy (±0.89), suggesting variability in performance due to possible redundancy, multicollinearity, and noise within the full set.

Applying MRMR with correlation filtering led to a reduction in dimensionality by nearly 49%, producing a refined subset of 222 features. While this resulted in a modest drop in testing accuracy to 89.52% and an *F*1-score of 82.48%, the trade-off was expected. The decline in performance likely reflects the removal of weakly correlated but complementary features, which, although individually insignificant, may contribute synergistically to classification in edge cases or transitional activities. Still, the MRMR configuration offered improved training efficiency and a narrowed feature space suitable for interpretability and downstream refinement.

In contrast, the RNCA configuration, consisting of only 83 features (a further 63% reduction from MRMR), achieved a training accuracy of 92.79% and a testing accuracy of 92.82%, surpassing both the full and MRMR models. It also delivered the highest *F*1-score of 86.91% and the narrowest confidence interval (±0.92), indicating both robust generalization and model stability. This result validates the effectiveness of the two-stage feature selection pipeline, where RNCA was able to fine-tune the MRMR-selected subset by assigning task-specific weights to each feature, effectively eliminating residual noise and redundancy. Several critical insights emerge from this comparative analysis:

- Simplicity without performance loss: The RNCA configuration used less than 20% of the original features yet outperformed or matched the full model in all key metrics. This underscores the principle that more features do not necessarily translate into better performance, especially in high-dimensional sensor data.
- Improved generalization: The narrower confidence intervals observed in the RNCA model indicate greater consistency across runs, a desirable property in real-world applications where model reliability is paramount.
- Computational efficiency: The reduced feature set not only simplifies the model structure making it easier to interpret but also decreases training and inference time, which is critical for deployment in resource-constrained environments or real-time sports analytics.

In summary, this evaluation illustrates that strategic feature selection first using MRMR for redundancy reduction and then RNCA for discriminative refinement can produce lightweight yet high-performing models. The RNCA configuration retained the most relevant biomechanical and statistical signals, enabling superior classification of complex human activity patterns in field hockey. This demonstrates that with appropriate dimensionality reduction strategies, high accuracy and robustness can be achieved without the computational burden of full-feature models.

### 4.4. Performance of Deep Learning Classifiers on Handcrafted Features

Table 3 illustrates the performances of all three deep learning models on testing dataset. It achieved comparable testing accuracies and macro *F*1-scores to the proposed hybrid feature-selection pipeline. Specifically, the RNCA configuration consistently yielded the highest classification performance across all architectures, achieving a mean testing accuracy of 94.13% ± 0.70 and a macro *F*1-score of 89.09% ± 1.70 with the MLP classifier. These findings confirm that the hybrid MRMR–RNCA framework effectively preserves the discriminative power of the original feature space while substantially reducing dimensionality from 432 to 83 features.

Among the evaluated architectures, the MLP consistently achieved the highest overall classification accuracy and macro *F*1-score across all feature configurations. Its performance advantage suggests that a fully connected structure is particularly well-suited for learning from static handcrafted features, where local temporal dependencies are absent. In contrast, the 1D-CNN and LSTM models, which are inherently designed for sequential or temporal data, exhibited weaker performance because the handcrafted features do not retain explicit temporal ordering or spatial structure for convolutional or recurrent operations to exploit.

The LSTM achieved moderately strong results compared to the CNN, indicating its partial ability to capture feature dependencies even in non-temporal representations. However, its overall accuracy remained below that of the MLP. Notably, for all three classifiers, the RNCA configuration yielded the most consistent results across runs, reflected by lower standard deviations in accuracy and *F*1-score. This consistency indicates that the RNCA refinement process not only reduced redundancy but also stabilized feature contributions, leading to improved generalization across different network architectures.

Furthermore, statistical analysis confirmed that the performance differences between the RNCA configuration and the full feature set were not statistically significant (*p* > 0.05) for MLP and CNN1D models, demonstrating that substantial feature reduction did not compromise classification accuracy. These findings collectively affirm that the hybrid MRMR–RNCA framework produces a compact, stable, and highly discriminative feature subset capable of supporting both classical and deep-learning-based classifiers with comparable or superior performance.

### 4.5. Confusion Matrix of RNCA

**Figure 4:**
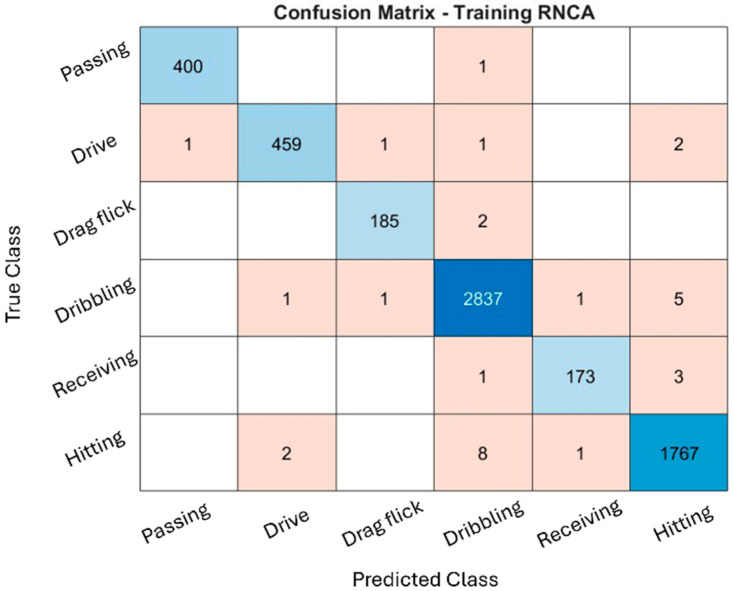
Training confusion matrix.

**Figure 5:**
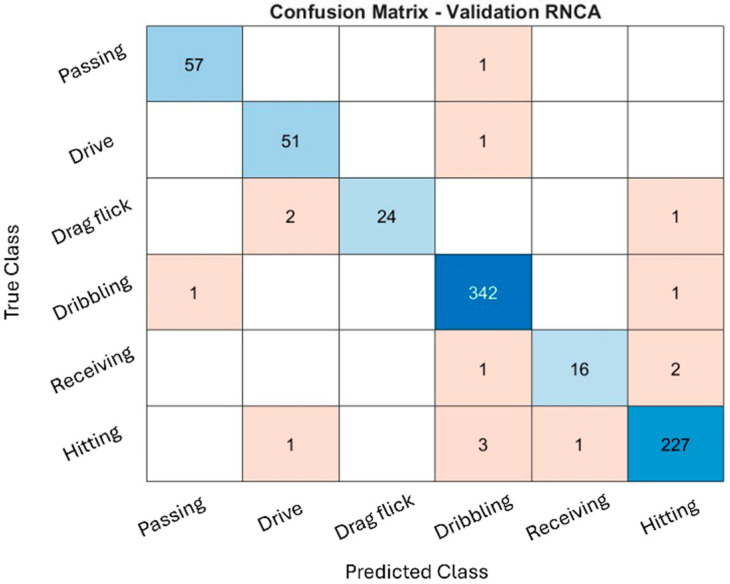
Validation confusion matrix.

**Figure 6:**
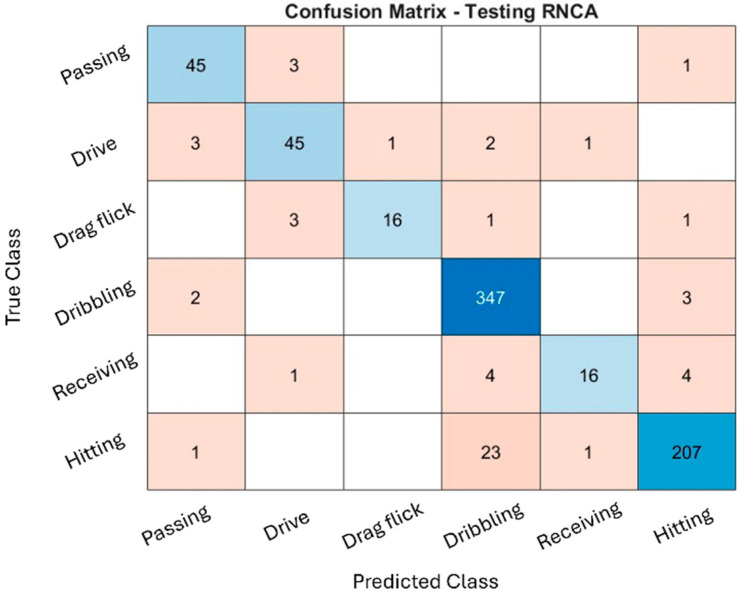
Testing confusion matrix.

The classification performance of the RNCA configuration was evaluated through confusion matrices across the training, validation, and testing datasets, as illustrated in Figure 4, Figure 5 and Figure 6. While the training and validation matrices are provided to visualize model learning behavior and class-specific distribution patterns, all performance metrics and generalization analyses in this study are based exclusively on the testing dataset to ensure fair and unbiased evaluation. These matrices provide deeper insight into how well the model distinguishes among specific field hockey activities: passing, drive, drag flick, dribbling, receiving, and hitting. The model achieved exceptionally high accuracy during training, particularly for dribbling and hitting, which recorded 2837 and 1767 correctly predicted samples, respectively. These activities are characterized by consistent and repetitive movement patterns, making them easier for the model to recognize. The distinct kinematic signatures, such as sustained acceleration and rotational motion captured by the sensors, likely contributed to their clear classification.

Minor misclassifications were observed. For instance, some hitting actions were misclassified as dribbling, likely due to shared upper-limb rotational patterns. Nevertheless, the overall training confusion matrix suggests that the model effectively captured the dominant biomechanical features associated with each activity without exhibiting signs of overfitting.

The validation confusion matrix reinforced the model’s generalization capability, with dribbling and hitting again achieving the highest classification performance (342 and 227 correct predictions, respectively). However, confusion increased for drag flick and receiving. Drag flick was frequently confused with passing and hitting, possibly due to similar preparatory postures or torso movements that occur across these techniques. Meanwhile, receiving, a motion characterized by subtle, short-duration kinetic activity, was often misclassified as dribbling or hitting. These overlaps highlight the biomechanical similarities between activities and the challenge of distinguishing them using only static, window-based features.

The performance remained consistent on the test set, with dribbling and hitting continuing to exhibit strong recognition (347 and 207 correct predictions, respectively). Misclassifications, however, were again prominent in drag flick and receiving, both of which were confused with multiple other activities. These patterns suggest that while the model is highly effective for distinct and repetitive actions, it faces challenges in recognizing brief, low-intensity, or context-dependent movements, which are more susceptible to misclassification due to overlapping inertial signatures. Additionally, variability in execution styles, sensor positioning, and lack of temporal continuity in the model may contribute to reduced performance for these complex activities.

The RNCA configuration exhibits strong accuracy for actions with clear biomechanical characteristics, such as dribbling and hitting, reflecting the success of the two-stage feature selection approach. However, recognition performance for drag flick and receiving is comparatively weaker, underscoring the limitations of using only static features for temporally nuanced or motion-overlapping activities. This confusion matrix analysis highlights both the strengths and limitations of the current approach and provides guidance for future refinement in sensor-based activity recognition classification.

### 4.6. Statistical Evaluation and Comparative Analysis—Paired t-Tests

Table 4 shows the comparative analysis between the Original Feature and MRMR configurations revealed a statistically significant difference in test accuracy with a *p*-value of less than 0.0001. This finding underscores the effectiveness of MRMR in reducing feature redundancy through its mechanism of selecting features that exhibit high relevance while maintaining minimal mutual information. However, the filter-based nature of MRMR inherently lacks context sensitivity. As a result, it may inadvertently exclude features that, although individually perceived as weakly relevant, may provide substantial contributions when considered in conjunction with other features. This oversight can lead to a notable decline in overall classification performance, as evidenced by the corresponding decrease in the *F*1-score. Furthermore, the performance contrast between the MRMR and RNCA configurations showed a statistically significant outcome as well, with a *p*-value also less than 0.0001. This finding emphasizes the advantages of utilizing supervised, embedded methods like RNCA for the refinement of features. Unlike MRMR, the RNCA approach learns joint feature weights that are informed by their collective contribution to minimizing classification error. This characteristic allows RNCA to retain features that are not just informative on an individual basis but also play an integral role in fostering complementary relationships with other features. This strategic retention mitigates the limitations observed with MRMR, thereby enhancing class separability and improving overall model performance.

Remarkably, the evaluation comparing the Original Feature and RNCA configurations revealed a *p*-value of 0.0589, which surpasses the Bonferroni-corrected significance threshold of α = 0.0167. This outcome suggests that there is no statistically significant difference in test accuracy between the two models. Practically, this indicates that the RNCA model achieves performance levels comparable to the Original Feature model, even with a considerably reduced feature set comprising only 83 features, merely 19% of the original 432. This significant feature dimensionality reduction reinforces the notion that a substantial portion of the original feature set likely consists of redundant or weakly informative attributes, which do not contribute meaningfully to predictive performance. In terms of confidence intervals, a detailed analysis of the 95% CIs for the test accuracies of each configuration further validates these Original Feature with accuracy of 92.24% with a confidence interval of [91.61%, 92.88%]—while the MRMR configuration yielded a lower accuracy of 89.52% with a wider interval of [88.76%, 90.28%]. The RNCA model achieved the highest mean accuracy of 92.82% and exhibited a similarly narrow confidence interval of [92.16%, 93.47%].

The broader confidence interval associated with the MRMR configuration suggests a higher level of variability and reduced stability in performance, likely attributed to the exclusion of synergistic features that could bolster predictive power. In contrast, the narrower confidence intervals observed for both the Original Feature and RNCA configurations indicate greater consistency and robust generalization performance. Notably, the RNCA model not only achieved the highest mean accuracy but also exhibited the lowest variability across different runs, thereby enhancing its reliability.

This statistical evaluation demonstrates that while the MRMR method effectively serves as an initial feature selection filter, its limitations in capturing intricate relationships among features diminish its standalone effectiveness. In contrast, the implementation of a two-stage hybrid approach that integrates MRMR with RNCA yields a model that is both compact and interpretable, while offering superior performance. The feature subset refined through RNCA performs comparably to the Original Feature configuration but achieves this with a significant reduction in dimensionality and computational demands. By retaining only the most informative features, this hybrid strategy facilitates scalable and practical deployment without sacrificing accuracy, thereby enhancing the overall effectiveness of the model.

### 4.7. Statistical Evaluation and Comparative Analysis—Correlation Heat Map

**Figure 7:**
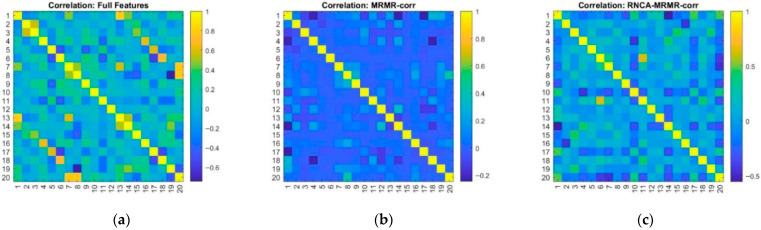
Influenced inter-feature dependencies and overall feature diversity for (**a**) all feature; (**b**) MRMR; and (**c**) RNCA.

Figure 7 illustrates Pearson correlation heatmaps for three distinct feature configurations. These heatmaps are integral in assessing linear inter-feature dependencies and evaluating the overall diversity of the selected features. In the Original Feature configuration, the heatmap presented in Figure 7a displays numerous bright yellow off-diagonal elements, which signify a substantial degree of linear correlation (|r| > 0.8) among various feature pairs. This correlation pattern is particularly pronounced among features derived from similar signal types, such as the means and variances measured by accelerometers along the same axis or originating from the same sensor location. The presence of such multicollinearity can have detrimental effects on certain classifiers by inflating the variance in coefficient estimation, thereby leading to model instability and a propensity for overfitting. This issue is especially pertinent in linear or distance-based models, such as k-NN, where reliance on correlated features can compromise the robustness and predictive performance of the classification model.

After implementing the MRMR with a correlation threshold set at *p* = 0.7, the results presented in Figure 7b indicate a notable reduction in the correlation among features. The majority of off-diagonal correlation values are now below the threshold of |r| < 0.3, signifying that the MRMR technique has effectively filtered out highly redundant features that do not contribute additional information. This outcome demonstrates the capacity of the algorithm to retain features that possess individual relevance while minimizing pairwise dependencies, thereby enhancing the statistical independence and diversity of the overall feature space. As a result, the model is strengthened in its robustness.

The heatmap corroborates this improvement by exhibiting a more diffuse and sparse structure, consistent with the primary objectives of the MRMR filtering approach which maximizing the relevance of retained features while minimizing redundancy. Subsequently, the RNCA configuration, as illustrated in Figure 7c, further refines the correlation structure. While RNCA does not specifically target the reduction in feature correlation, the synergistic combination of supervised feature weighting and prior MRMR filtering has led to a feature set that is even more decorrelated. The remaining features present varying degrees of correlation, predominantly showing minimal to moderate correlations, as indicated by the darker cells in the correlation matrix.

Notably, some feature pairs continue to exhibit modest correlations. This may reflect a deliberate decision to retain certain synergistic features that mutually enhance discriminative value of one another, thereby enriching the predictive capabilities of the model. This strategy aligns with objective of RNCA to optimize classification performance, prioritizing model effectiveness even at the expense of allowing slight inter-feature dependencies.

Overall, the observed patterns underscore the complementary roles of MRMR and RNCA within a hybrid feature selection pipeline. MRMR acts as a stringent linear redundancy filter that ensures only the most relevant and non-redundant features are retained. Conversely, RNCA adaptively retains feature combinations that maximize class separation, even if such retention entails minor redundancies. Together, these methodologies result in a compact, decorrelated feature set that contributes to achieving high predictive performance while efficiently addressing the risk of overfitting within the model.

### 4.8. Statistical Evaluation and Comparative Analysis—PCA and t-SNE

**Figure 8:**
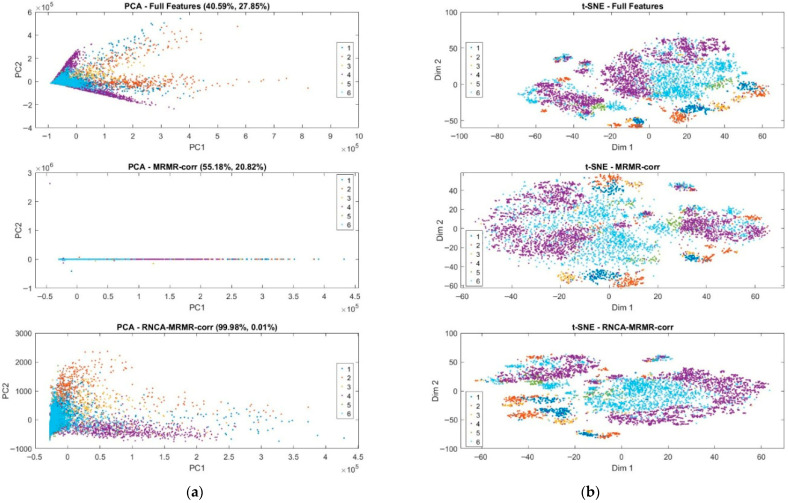
Dimensionality reduction using (**a**) PCA and (**b**) t-SNE.

The visual results of dimensionality reduction achieved through PCA and t-SNE across three distinct feature configurations. In the original features configuration, PCA yielded a triangular dispersion pattern in the PC1–PC2 space, where the two principal components account for 40.59% and 27.85% of the total variance, respectively, as depicted in Figure 8. This moderate coverage of variance advocates the presence of substantial redundancy and noise within the original set of 432 features. The extensive spread and notable overlap observed among different activity clusters imply that linear separability is limited, thereby complicating the differentiation between distinct activity classes based solely on these principal components. Moreover, this conclusion is reinforced by the findings from the t-SNE visualization, which displayed only loosely defined clusters characterized by considerable overlap, as illustrated in Figure 8b. The scattered distribution evident in the t-SNE plot indicates the existence of nonlinear relationships among the features; however, these relationships remain inadequately captured by the high-dimensional representation of the Original Features configuration. Consequently, the visual outcomes from both PCA and t-SNE underscore the necessity for more refined feature selection techniques to enhance the representation of class structures in the analyzed data.

In contrast, the MRMR configuration exhibited a significant enhancement in the PCA representation. Specifically, the first principal component accounted for 55.18% of the total variance, underscoring that the process of eliminating redundant features through MRMR filtering has allowed more informative variance to become predominant within the dataset. However, it is noteworthy that the PCA projection displayed a pronounced skew along a linear axis, resulting in a limited spread in the second principal component (PC2), which accounted for only 20.82% of the variance. This limited spread ultimately led to reduced interpretability of the data. The observed distortion may be attributed to a few dominant features exerting a disproportionate influence on the overall variance structure, thereby overshadowing less prominent yet potentially informative features.

Conversely, the t-SNE projection provided a more insightful representation of the data. When compared to the original feature configuration, the t-SNE analysis revealed markedly clearer class boundaries, characterized by diminished inter-class overlap. This improvement highlights the efficacy of the MRMR method in selecting discriminative features that substantially enhance nonlinear class separability. The ability of t-SNE to maintain local structures while revealing global data organization further emphasizes the value of MRMR in facilitating robust feature selection, ultimately contributing to the advancement of classification tasks in complex datasets.

The RNCA configuration yielded compelling results, demonstrating that Principal Component 1 (PC1) accounted for 99.98% of the overall variance, while Principal Component 2 (PC2) explained a mere 0.01%. This disproportionate distribution suggests that the final refined feature set is heavily influenced by a limited number of highly weighted features. Such an outcome underscores the optimization strategy employed by RNCA, which prioritizes class-discriminative features through a supervised learning framework. However, this extreme concentration of variance rendered the PCA plot effectively one-dimensional, subsequently diminishing its interpretability.

Conversely, the corresponding t-SNE visualization provided the most distinct and compact class clusters across all configurations analysed. The observed clear separability and tighter groupings serve to illustrate that RNCA has successfully maintained the local neighborhood structures that are essential for precise classification. These findings elucidate the complementary strengths inherent in PCA and t-SNE. PCA serves a dual purpose: it quantifies the variance attributed to the selected features and reveals linear relationships between them. In contrast, t-SNE excels in capturing the intricate nonlinear interactions that delineate class boundaries. The progressive enhancement in cluster definition observed in the t-SNE plots moving from the original feature set to MRMR and culminating in RNCA provides compelling evidence for the effectiveness of feature selection pipeline in refining class discriminability

Hence, the analysis of feature dimensionality reduction confirms that the integration of MRMR correlation with RNCA not only streamlines the feature space but also significantly improves class separability in both linear and nonlinear contexts. This validates the efficacy of proposed hybrid approach in optimizing feature representations, thus ensuring robustness and interpretability in field hockey classification utilizing from inertial sensor data.

### 4.9. Computational Complexity Analysis

Table 5 presents a comprehensive summary of the average training and inference times obtained from ten independent runs. The results demonstrate that dimensionality reduction markedly decreases computational cost across all classifiers. When utilizing the original feature set containing 432 features, the training process required an average of 4.96 ± 0.63 s, with an inference time of 0.296 ± 0.016 s. Applying the MRMR configuration, which reduced the feature set to 222 features, substantially decreased training time to 2.67 ± 0.17 s (a reduction of 46.22%) and inference time to 0.157 ± 0.005 s (a reduction of 46.99%). The most efficient configuration was achieved using the RNCA algorithm, which further condensed the feature set to 83 features. This approach reduced training time to 1.28 ± 0.07 s (−74.22%) and inference time to 0.060 ± 0.002 s (−79.85%), corresponding to 3.9-fold faster training and 5.0-fold faster inference compared with the full-feature model.

On average, the deep learning classifiers required approximately three- to six-times longer training durations than the proposed MRMR–RNCA pipeline yet produced only marginal accuracy improvements. These results highlight the significant computational advantage of the hybrid feature-selection framework. By substantially reducing dimensionality while maintaining predictive accuracy, the proposed approach offers a more lightweight and interpretable alternative to deep neural models.

The observed reductions in both training and inference times confirm the framework’s suitability for real-time and resource-constrained deployments, such as wearable or embedded sports-performance monitoring systems. Overall, these findings emphasize the practical value of integrating dimensionality-reduction strategies into HAR classification workflows. This enables efficient, high-performance analytics for field hockey and similar sensor-based sports activity recognition.

## 5. Conclusions

This study presented a hybrid feature-selection framework that integrates MRMR with RNCA to enhance the classification of sport-specific human activities from inertial sensor data in field hockey. A comprehensive feature extraction stage yielded 432 time- and frequency-domain features per sliding window from four strategically placed IMUs. Given this high dimensionality, feature selection was essential to improving generalization, computational efficiency, and interpretability.

In the first stage, MRMR combined with Pearson correlation filtering (|*p*| > 0.7) eliminated redundant and highly correlated features, reducing the set from 432 to 222 while emphasizing statistical independence. In the second stage, RNCA assigned supervised weights to maximize discriminative power, further reducing the dimensionality to 83 features with approximately 19% of the original feature space while retaining the most salient information for classification. The results confirmed the effectiveness of this two-stage approach. The RNCA configuration achieved a testing accuracy of 94.13% ± 0.70 and a macro *F*1-score of 89.09% ± 1.70, surpassing the MRMR-only configuration (92.84% ± 0.70, 86.26% ± 1.80) and slightly outperforming the full-feature baseline (93.91% ± 0.80, 87.97% ± 2.50). These findings demonstrate that the hybrid MRMR–RNCA pipeline preserved predictive performance while achieving a substantial reduction in feature dimensionality.

Moreover, this study provides a broader comparison using several deep learning classifiers. MLP, 1D-CNN and LSTM were trained using the same handcrafted feature sets. The RNCA configuration consistently produced the highest performance across all three architectures, confirming the robustness and generality of the selected feature subset. Despite the increased complexity of deep networks, their performance was comparable to or slightly lower than that of the proposed hybrid pipeline, reaffirming the framework’s efficiency and interpretability.

Beyond predictive accuracy, the hybrid framework delivered substantial computational efficiency. Compared with the full-feature model, training time decreased by approximately 74% and inference time by 80%, corresponding to a 3.9-fold and 5.0-fold improvement, respectively. These improvements demonstrate the practicality of the proposed method for real-time and resource-constrained applications, such as wearable sports monitoring systems. Moreover, feature-weight analysis revealed that accelerometer-derived features, particularly along the *X* and *Y* axes, contributed most strongly to classification, suggesting that translational motion plays a key role in distinguishing field hockey actions.

Despite these contributions, several limitations must be acknowledged. Both MRMR and RNCA rely on linear assumptions, potentially overlooking nonlinear dependencies present in complex or noisy datasets. The current framework also operates on static, windowed features without modeling temporal transitions between activities. Furthermore, validation was limited to a single sport-specific dataset, constraining generalization to broader contexts.

Future study should extend this framework by integrating temporal deep learning architectures, for instance, LSTMs, attention-based models, or transformers that capable of capturing sequential dependencies in raw IMU signals, and by exploring nonlinear feature-selection strategies such as mutual-information maximization, kernel-based methods, or manifold learning. Additionally, evaluating the framework under LOSO and cross-dataset validation protocols will further substantiate its generalization across individuals and activity domains.

In conclusion, the proposed MRMR–RNCA hybrid pipeline offers an interpretable, compact, and computationally efficient solution for sensor-based human activity recognition. By balancing redundancy elimination with supervised feature weighting, it achieves high performance with substantially fewer features, outperforming more complex deep learning classifiers under equivalent feature conditions. The framework thus provides a practical and scalable framework for future HAR pipelines in sports analytics, rehabilitation, and wearable monitoring, combining methodological rigor with real-sport applicability.

## Figures and Tables

**Table 1:** Top 10—features with Score.

Rank	Feature Rank by MRMR	Score
1	Wrange_gyroZ2	0.330704
2	Wmean_accY4	0.327858
3	Wvar_accY3	0.219757
4	Wmedian_gyroZ1	0.219757
5	Wskewness_gyroX4	0.161169
6	Wharmmean_gyroX2	0.009665
7	Wpowerbw_gyroX2	0.003708
8	Wkurtosis_gyroZ1	0.002643
9	Wpowerbw_accX1	0.002067
10	Wmedian_accZ4	0.001973

**Table 2:** Accuracy of models for different configurations.

Model	Training Accuracy/*F*1 Score	Validation Accuracy/*F*1 Score	Testing Accuracy/*F*1 Score
Original (432 features)	92.32% (±0.30) 86.55 (±0.51)	92.90% (±1.09) 87.05 (±2.66)	92.24% (±0.89) 85.84 (±1.61)
MRMR (222 features)	89.13% (±0.25) 82.53 (±0.52)	89.71% (±0.89) 83.00 (±1.98)	89.52% (±1.06) 82.48 (±2.11)
RNCA (83 features)	92.79% (±0.22) 87.35 (±0.40)	92.61% (±0.70) 86.78 (±2.08)	92.82% (±0.92) 86.91 (±1.67)

**Table 3:** Accuracy of models for different configurations.

Model	Classifier	Accuracy (%)	*F*1-Score (%)
Original	MLP	93.91 ± 0.80	87.97 ± 2.50
	1D-CNN	69.93 ± 1.20	45.69 ± 3.60
	LSTM	76.64 ± 1.30	63.78 ± 2.30
MRMR	MLP	92.84 ± 0.70	86.26 ± 1.80
	1D-CNN	64.51 ± 1.70	35.85 ± 2.10
	LSTM	56.20 ± 4.60	22.98 ± 6.40
RNCA	MLP	94.13 ± 0.70	89.09 ± 1.70
	1D-CNN	70.45 ± 1.30	52.24 ± 1.80
	LSTM	78.27 ± 1.40	63.43 ± 3.00

**Table 4:** Paired *t*-test of different configurations.

	*t*-Test
Original Feature vs. MRMR	*p* = 0.0001
Original Feature vs. RNCA	*p* = 0.0589
MRMR vs. RNCA	*p* = 0.0000

**Table 5:** Computational Running time.

	K-NN		MLP		1D-CNN		LSTM	
Model	Training Time (s)	Inference Time (s)	Training Time (s)	Inference Time (s)	Training Time (s)	Inference Time (s)	Training Time (s)	Inference Time (s)
Original features	4.9569 ± 0.6276	0.2962 ± 0.0156	106.63 ± 21.36	0.00006 ± 0.00005	228.01 ± 41.45	0.00019 ± 0.00003	306.25 ± 76.28	0.00027 ± 0.00010
MRMR	2.6656 ± 0.1743	0.1570 ± 0.0050	99.77 ± 25.00	0.00004 ± 0.00001	155.99 ± 39.68	0.00014 ± 0.00001	199.45 ± 27.51	0.00019 ± 0.00001
RNCA	1.2781 ± 0.0737	0.0597 ± 0.0022	106.37 ± 14.23	0.00003 ± 0.00001	181.74 ± 6.95	0.00010 ± 0.00002	144.27 ± 35.68	0.00010 ± 0.00003

## Data Availability

The data presented in this study are currently under further analysis and are not publicly available at this time. However, the dataset can be made available by the corresponding author upon reasonable request, subject to ethical approval and compliance with data protection guidelines.

## Abbreviations

The following abbreviations are used in this manuscript:

IMU	Inertia measurement unit
AI	Artificial intelligence
MRMR	Minimum redundant maximum relevant
NCA	Neighborhood component analysis
RNCA	Regularized neighborhood component analysis
